# Gene Mapping and Genetic Analysis of Maize Resistance to Stalk Rot

**DOI:** 10.3390/ijms262411866

**Published:** 2025-12-09

**Authors:** Baobao Wang, Shaoxin Wang, Luo Xu, Zhongjian Li, Xin Ding, Liang Ma, Zixiang Cheng, Jianying Feng, Canxing Duan

**Affiliations:** 1 Shijiazhuang Academy of Agriculture and Forestry Sciences, Shijiazhuang 050041, China; 15732258101@163.com (B.W.); wangshaoxin666@163.com (S.W.); sjzxuluo@163.com (L.X.); lzhj888@163.com (Z.L.); 13231156822@163.com (X.D.); 13785201176@163.com (L.M.); 2 State Key Laboratory of Crop Gene Resources and Breeding, Institute of Crop Sciences, Chinese Academy of Agricultural Sciences, Beijing 100081, China; chengzixiang@caas.cn

**Keywords:** maize, *Fusarium graminearum*, stalk rot, BSA-seq, candidate genes

## Abstract

Fusarium stalk rot, which is a common disease caused by *Fusarium* species, can seriously decrease maize grain yield and quality. Hence, the genetic mechanism mediating maize resistance to Fusarium stalk rot must be elucidated and the associated resistance genes useful for breeding disease-resistant cultivars should be identified. In this study, the highly resistant maize inbred line H1710 and highly susceptible inbred line Huangzaosi were used to construct segregating populations through hybridization, backcrossing, and other methods. A resistance/susceptibility pool was constructed from the F_2_ population. A BSA-seq analysis revealed one candidate region associated with stalk rot resistance on chromosome 6; this region (3.98 Mb) contains 38 genes. Furthermore, KASP molecular markers designed for the candidate region precisely localized two candidate genes, *Zm00001eb260120* and *Zm00001eb260260*, which were considered to be the most likely genes mediating stalk rot resistance. The study findings lay a theoretical foundation for analyzing the molecular basis of maize resistance to Fusarium stalk rot and cloning the relevant resistance genes.

## 1. Introduction

Maize (*Zea mays* L.) is one of the most important cereal crops cultivated worldwide; it is responsible for approximately 12% (1.2 billion tonnes) of global grain production, with growth rates that were 2.6 to 3.3 times higher than those of wheat and rice in 2020–2022 [1]. Maize stalk rot, a soil-borne disease prevalent in maize-producing regions worldwide, is a major threat to sustainable crop yields. This disease is caused by various fungi and bacteria, including more than 30 types of pathogenic fungi. In China, the primary pathogens causing stalk rot are *Pythium inflatum*, *Pythium graminicola* (class Oomycetes) [2], and *Fusarium graminearum* [3]. By contrast, internationally, *Fusarium verticillioides* and *F. graminearum* are the main pathogens responsible for stalk rot [4]. Fusarium stalk rot (FSR) typically decreases maize yields by 10%, reaching 30–50% in severely affected areas in China [5]. FSR can cause maize yield losses of 38–100% in the United States and Canada, but it may also result in grains and plants contaminated by low-molecular-weight secondary metabolites known as mycotoxins, which pose severe risks to human and animal health [6]. Maize plants affected by premature stalk rot produce lightweight, poorly filled ears because of limited carbohydrate availability during the grain-filling stage. Infected stalks deteriorate from sturdy, solid rods to hollow tubes as the stalk pith separates from the outer rind, thereby compromising structural integrity. These weakened stalks are susceptible to lodging, particularly when the decay occurs below the ear [7]. Identifying disease resistance genes and enhancing maize stress resistance represent crucial approaches to mitigating the harmful effects of stalk rot. Traditional methods for controlling stalk rot, including chemical treatments and agronomic practices, are inefficient and costly, emphasizing the need for sustainable solutions involving genetic resistance.

Multiple factors influence stalk rot symptom development, including pathogen type, environmental conditions, and plant growth status. The resistance of maize to Fusarium stalk rot typically manifests as a quantitative trait. Quantitative trait locus (QTL) mapping has been used to identify genetic regions associated with stalk rot resistance in maize [8]. Chen et al. [9] conducted a QTL-seq analysis using F_2_ and BC_1_F_1_ populations derived from the 18,327 × S72356 cross to map a QTL (*Rgsr8.1*) for resistance to stalk rot caused by *F. graminearum* on chromosome 8 (161.001—170.6 Mb). On the basis of simple sequence repeat (SSR) markers, single-nucleotide polymorphism (SNP) markers, and recombination detection, they more precisely mapped *Rgsr8.1* to a 2.04 Mb region. Liu et al. [10] conducted a genome-wide association study (GWAS) involving 165 inbred lines and identified 34 SNPs significantly associated with stalk rot resistance. Subsequent linkage mapping validated *GRMZM2G096904* and *GRMZM2G010912* as candidate resistance genes. In another GWAS, Song et al. [11] analyzed seedling blight resistance using 219 inbred lines. Notably, they identified *ZmSBR1* as a candidate gene significantly associated with maize seedling resistance to *Exserohilum turcicum*. A phenotypic analysis of EMS mutant lines indicated that *ZmSBR1* also confers resistance to *F. graminearum* stalk rot in adult plants. The antioxidant protein ZmPrx5 also contributes to stalk rot resistance [12]. Bocianowski et al. [13] applied NGS technology and association mapping to identify candidate genes for FSR resistance; they detected marker 4,772,836 within the serine/threonine protein kinase gene *bsk3* and marker 4,765,764 within the histidine kinase 1 gene. These genes associated with plant resistance to FSR are potentially useful for breeding disease-resistant varieties.

Tropical maize germplasm resources are more resistant to stalk rot than other genetically similar maize germplasms [14]. Late-maturing varieties are typically more resistant to stalk rot than early-maturing varieties [15]. Moreover, disease resistance varies among maize germplasm resources belonging to different genetic groups. Inbred lines from the Dalian/Jilin red-kernel varieties and selected lines of foreign hybrid varieties, such as Lancaster, Reid, and P groups in the US, carry many stalk rot resistance-related genes. However, germplasm resources in the Tangsi Pingtou group, which is represented by Huangzaosi, are mostly susceptible to stalk rot, with relatively few resistance-related genes. Multi-generational breeding at the Shijiazhuang Academy of Agriculture and Forestry Sciences has resulted in the development of the backbone inbred line H1710. Specific traits of this line (e.g., disease resistance, a stay-green phenotype, wide adaptability, and a strong combining ability) have been exploited to generate stalk rot-resistant maize varieties, including DefengC919, Shiyu15, Defeng Jinyu5, Shiyu191, Shiyu193, and Jingshi 211. Although H1710 exhibits comprehensive disease resistance (e.g., stalk rot resistance) and lodging resistance, the mechanism underlying its disease resistance remains unclear. In this study, a BSA-seq analysis was combined with field experiments to characterize the stalk rot resistance mechanism and identify key resistance genes, thereby providing a theoretical foundation for breeding stalk rot-resistant maize varieties.

## 2. Results

### 2.1. Infection of Maize by F. graminearum

Previous research has demonstrated that maize inbred lines Huangzaosi and H1710 are, respectively, susceptible and highly resistant to stalk rot, with Huangzaosi being susceptible to a disease severity score of 8.9 and H1710 having a disease severity score of 1.6.

### 2.2. Phenotypic Identification of Maize Stalk Rot Resistance

**Figure 1:**
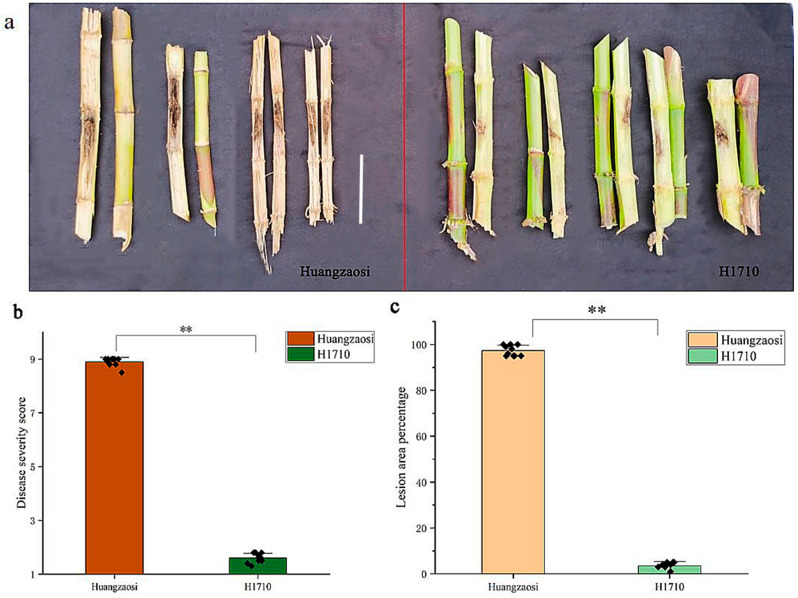
(**a**) H1710 is more resistant to stalk rot than Huangzaosi. Representative examples of infected Huangzaosi and H1710 samples are presented on the left and right, respectively. (**b**) Disease severity score of Huangzaosi and H1710. (**c**) Lesion area percentage of Huangzaosi and H1710. ** statistically significant (*p* < 0.01) between mock and inoculation treatments analyzed by *t*-test. The white scale bar represents 10 cm.

A comparison of artificially inoculated H1710 and Huangzaosi revealed that the lesion area percentages were 0% to 5% and 95% to 100%, respectively (Figure 1).

### 2.3. BSA-Seq Data Analysis

#### 2.3.1. Identification of QTLs Related to Stalk Rot Resistance via BSA-Seq

**Figure 2:**
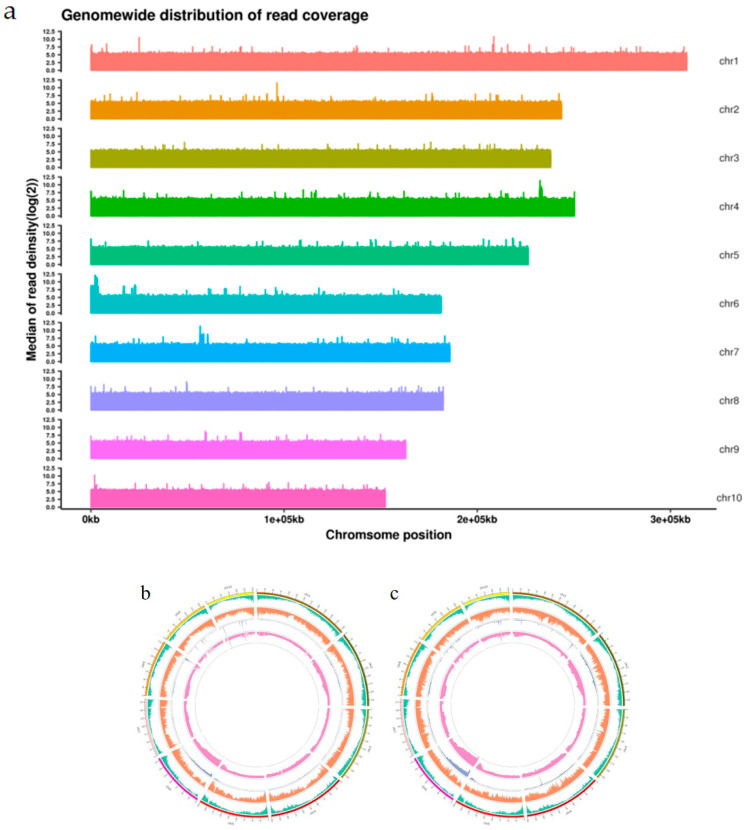
(**a**) Genome-wide distribution of reads. (**b**) Visualization of the chromosomal distribution of InDels. The five circles represent the following (from outside in): chromosomal coordinates, gene distribution, InDel density distribution, InDel-ED value distribution, and ΔInDel index value distribution. (**c**) Visualization of the chromosomal distribution of SNPs. The five circles represent the following (from outside in): chromosomal coordinates, gene distribution, SNP density distribution, SNP-ED value distribution, and ΔSNP index value distribution.

Data quality control: Sequencing yielded 153.37 Gbp of clean data, with Q30 reaching 94.97% (Supplementary S1, Table S1 and Supplementary S2, Figure S1). An average of 98.86% of the clean reads (Supplementary S1, Table S2 and Supplementary S2, Figure S2) were aligned to the reference genome, with an average coverage depth of 16.50× and genome coverage of 91.37% (Supplementary S1, Table S3) (at least one base covered) (Figure 2a).

Mutation detection: A total of 5,958,224 SNPs were detected between parents, including 66,990 non-synonymous SNPs (Supplementary S1, Table S4 and Supplementary S2, Figure S3). Additionally, 2,040,838 SNPs were detected between the pooled samples (Supplementary S2, Figure S4-1), with 9869 resulting in non-synonymous mutations (Figure 2b,c). Furthermore, 872,183 and 273,665 small insertion/deletions (InDels) were detected between the parents and between the pooled samples, respectively (Supplementary S2, Figure S4-2).

#### 2.3.2. Association Analyses Using the ED Association Algorithm and Index Association Algorithm

**Figure 3:**
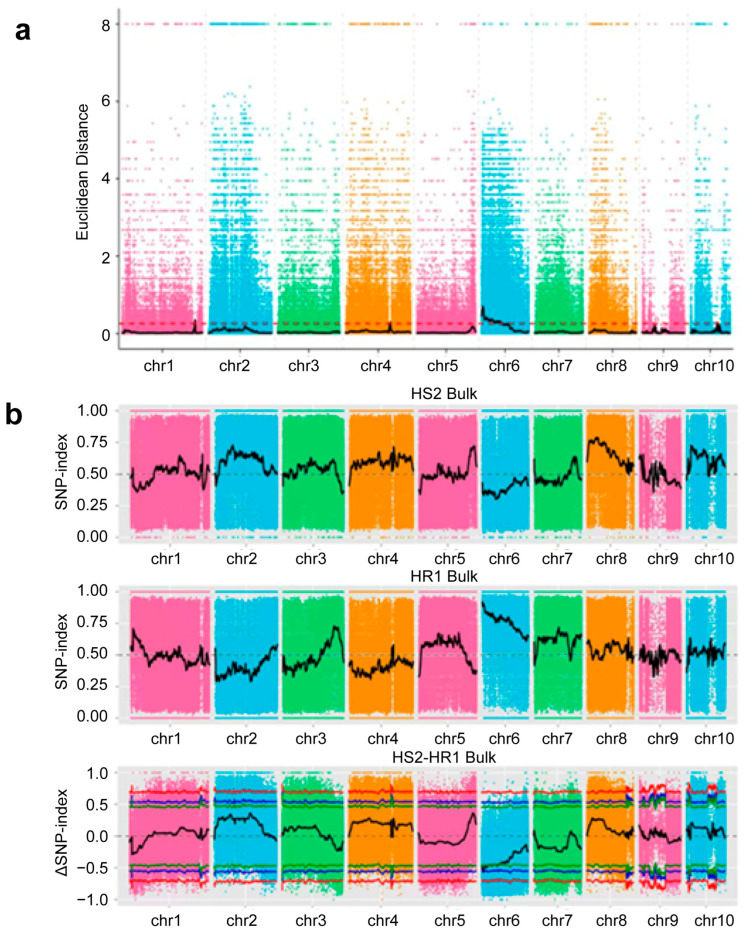
(**a**) Chromosomal distribution of ED association values (SNP). (**b**) Chromosomal distribution of SNP index association values. In the figure, the red line represents the 99% confidence interval, the blue line represents the 95% confidence interval, and the green line represents the 90% confidence interval.

SNP results: The ED association algorithm identified 20 candidate regions (with a total length of 83.70 Mb) associated with stalk rot resistance. The SNP-index association algorithm detected one candidate region (3.96 Mb) related to stalk rot resistance. On the basis of the intersecting results of these two methods, a candidate region associated with stalk rot resistance was revealed (3.96 Mb) (Figure 3a,b).

**Figure 4:**
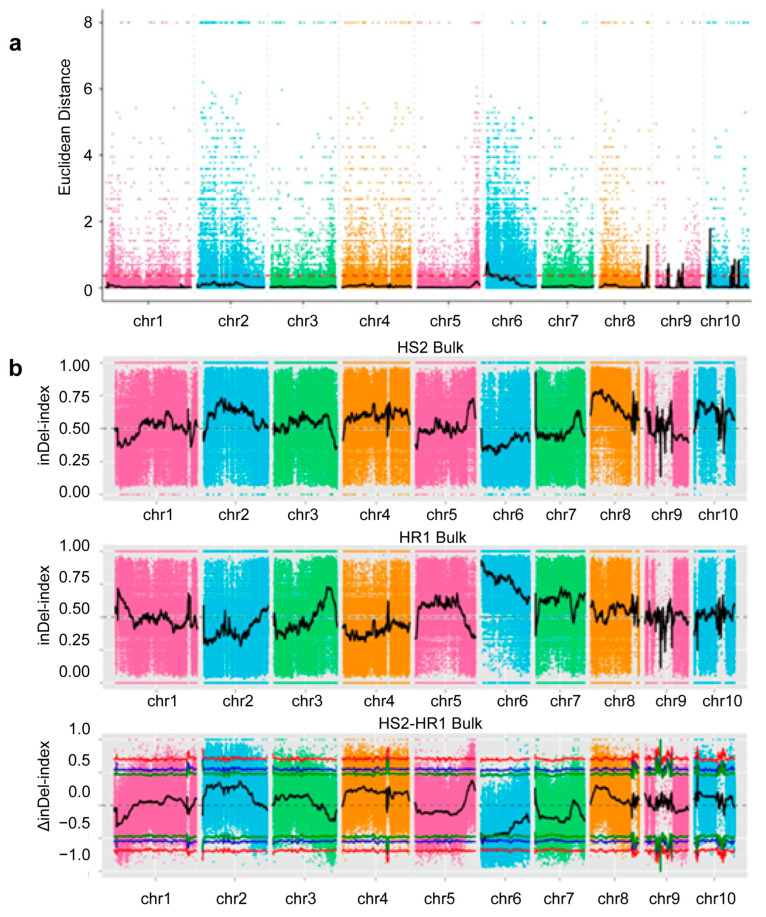
(**a**) Chromosomal distribution of ED association values (InDel). (**b**) Chromosomal distribution of InDel index association values. In the figure, the red line represents the 99% confidence interval, the blue line represents the 95% confidence interval, and the green line represents the 90% confidence interval.

InDel results: The ED association algorithm identified 37 candidate regions (with a total length of 72.60 Mb) related to stalk rot resistance. The InDel-index association algorithm also identified a candidate region (6.86 Mb) related to stalk rot resistance. According to the intersecting results of these two methods, a candidate region related to stalk rot resistance was detected (6.86 Mb) (Figure 4a,b).

**Figure 5:**
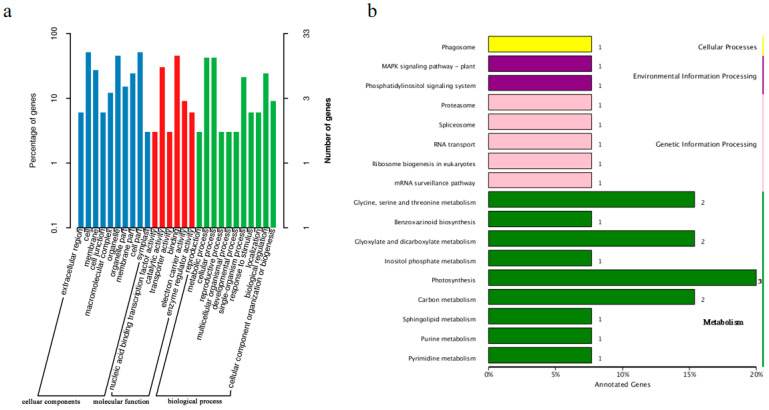
(**a**) GO annotations of genes in the candidate region. The horizontal axis presents GO categories. The vertical axes on the left and right present the percentage of genes in each category and the actual number of genes in each category, respectively. Genes within the candidate region were classified using all genes as the background. (**b**) Annotation of genes in the candidate region according to KEGG pathways. The horizontal axis presents the number of genes and the proportion of all annotated genes assigned to a particular pathway. The vertical axis presents KEGG metabolic pathways.

Intersecting results for SNP and InDel analyses: One candidate region related to stalk rot resistance spanning 3.96 Mb (3,280,000–7,240,000 bp) was identified on chromosome 6. This region contained 38 genes, including 21 genes with non-synonymous mutations and 3 genes with frameshift mutations between the parental materials (Figure 5a) (Supplementary S1, Table S5).

### 2.4. Annotation of Genes in the Candidate Region

BLAST and MaizeGDB were used to annotate genes in the candidate region (3.96 Mb) on chromosome 6 on the basis of the NR, Swiss-Prot, GO, KEGG, and COG databases (Supplementary S1, Tables S6–S10). These genes were classified by assigning them to specific KEGG pathways (Figure 5b).

### 2.5. Competitive Allele-Specific PCR Assay

**Figure 6:**
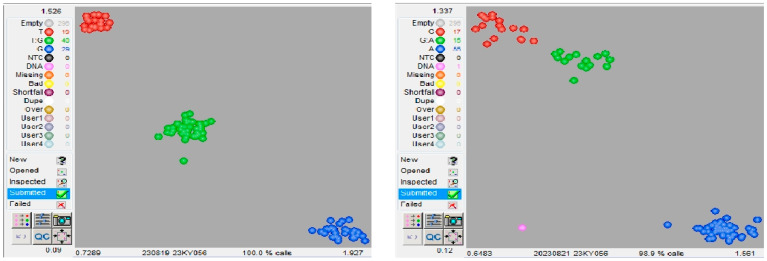
KASP-based genotyping using primers sn6640608 and sn7185874.

Competitive allele-specific PCR (KASP) primers (Supplementary Table S1) were designed for SNPs or InDels in candidate genes for stalk rot resistance in maize. KASP molecular markers were designed for candidate region genes with non-synonymous mutations (Table 1). More than 80 high-yielding materials (Supplementary S2, Table S5) that were highly susceptible to stalk rot were screened from more than 600 materials in the F_2:3_ population derived from the cross between Huangzaosi and H1710. These materials were analyzed using the designed KASP molecular markers. The electrophoresis results indicated that two pairs of molecular markers corresponded to 60–70% of the observed phenotypes (Figure 6). Stalk rot severity differed significantly between allele genotypes at SNP2 and SNP10 loci. SNP2 (T or G) was located at position 6,640,608 on chromosome 6. Among 85 maize-derived materials, 29 had the genotype GG at the SNP2 locus, with an average stalk rot severity score of 7.8. By contrast, 19 materials had the genotype TT at this locus, with an average stalk rot severity score of 2.1. SNP10 (A or G) was located at position 7,185,874 on chromosome 6. Among the 85 maize-derived materials, 17 had the genotype GG at the SNP10 locus, with an average stalk rot severity score of 5.8, whereas 55 materials had the genotype AA at this locus, with an average stalk rot severity score of 4.5. Using MaizeGDB, the gene containing SNP2 was identified as *Zm00001eb260120* (chromosome 6:6,637,440–6,641,806 for Zm-B73-REFERENCE-NAM-5.0), which encodes protein 2, containing the receptor homology region transmembrane domain and RING domain. The gene corresponding to SNP10 was identified as *Zm00001eb260260*, which encodes an S-adenosyl-L-methionine-dependent methyltransferase superfamily protein that catalyzes the transfer of a methyl group to an acceptor molecule.

## 3. Discussion

Stalk rot is a soil-borne disease caused by several pathogens. *F. graminearum*, which is an important fungal pathogen causing FSR in maize, is a hemibiotroph that infects roots (i.e., it is a soil-borne inoculum) and spreads throughout the infected plant via nodes and other aerial parts. Maize stems affected by stalk rot contain large quantities of toxins, making roughage prepared from infected maize stalks potentially unsafe. The presence of toxigenic fungi from the genus *Fusarium* can adversely affect grain yield and quality [16]. Therefore, controlling stalk rot is critical for stable maize production. Selecting superior resistant germplasm resources and breeding resistant varieties is the most economical and effective strategy for controlling stalk rot.

Several common artificial inoculation methods have been used to identify and evaluate global maize germplasm resources for stalk rot resistance. However, the utility of the toothpick, ball bearing, and root infection methods is limited by the inability to control and monitor inoculum dosage. Moreover, oat kernel inoculation is constrained by inconsistent and/or low FSR infection rates across genotypes, likely because of variability in terms of fungal colonization [17]. In the current study, a needle injection-based inoculation method was implemented to evaluate quantitative FSR resistance because it is a widely used method for delivering precise and adjustable amounts of *Fusarium* inoculum (i.e., spores). This approach offers significant advantages over natural infection in the field, primarily by ensuring uniform and reproducible disease pressure across all experimental plants. By effectively bypassing the unpredictable environmental barriers inherent to field conditions, such as fluctuating humidity and temperature, this method allows for a more accurate and reliable assessment of the host’s genetic resistance, thereby minimizing experimental noise and enhancing the resolution of quantitative trait mapping [18].

Research involving the mapping of maize genes related to stalk rot resistance has been conducted since the 1990s. Several factors, including pathogen type, environmental conditions, and plant status, affect stalk rot symptom development. Host genetic resistance to stalk rot is considered to be a qualitative and quantitative trait. The mapping of genes associated with stalk rot resistance has typically focused on stalk rot caused by individual pathogens, primarily *F. verticillioides*, *Pythium* species, and *F. graminearum*. Linkage mapping is useful for examining recombination events and marker–trait associations in bi-parental segregating populations (e.g., F_2_, doubled haploid, and recombinant inbred lines), while also providing significant advantages over other methods for detecting QTLs. Other approaches, such as BSA-seq, genome-wide association analysis [19], and transcriptome analysis [20], have been used to identify disease resistance genes. In this study, we identified candidate regions associated with stalk rot resistance via BSA-seq, which is a forward genetics-based technique appropriate for identifying and regulating target traits. For crops with sequenced reference genomes, BSA-seq can be used to locate candidate regions for traits controlled by one or two genes, as well as quantitative traits governed by major genes and polygenes [21]. This technique has been used extensively to map maize genes. By integrating BSA-seq, RNA-seq, and phytohormone analyses, Yan et al. [22] determined that the fasciated ear phenotype of a novel maize mutant is likely due to a recessive mutation in *Zm00001d048841*.

We functionally annotated genes within the identified candidate region using bioinformatics methods and then developed KASP molecular markers to precisely localize genes on specific chromosomes. KASP is a homogeneous, fluorescence-based, endpoint genotyping technology that represents the most straightforward, cost-effective, and flexible approach to determining SNP and InDel genotypes [23]. Notably, although KASP molecular markers were used to precisely localize candidate genes, during the subsequent verification by other groups, the KASP molecular markers did not demonstrate complete one-to-one correspondence with phenotypes. Multiple factors may have contributed to this outcome. For example, sequences near the SNP may have hindered PCR amplifications using the designed primers or template DNA quality requirements were not satisfied. To enhance the utility of KASP technology, future studies should focus on identifying and summarizing functional gene sites relevant to basic research and establishing a KASP database of related functional gene polymorphic sites. Additionally, to improve the detection of unknown samples, standard positive control samples should be established as functional KASP markers are developed. Under laboratory-based small-scale detection conditions, template DNA quality must be maintained, and similar concentrations should be used. A KASP assay developed on the basis of SNP markers closely linked to stalk rot resistance is potentially relevant to developing practical high-throughput PCR assays for screening maize breeding populations for stalk rot resistance.

Previous studies identified several FSR resistance loci, including five QTLs [24], *Rfg1* [25], *qRfg1* and *qRfg2* [26], and *qRfg3* [27]. In addition, Chen et al. [9] used a QTL-seq method and F_2_ and BC_1_F_1_ populations derived from a cross between 18,327 and S72356 to map a QTL (*Rgsr8.1*) for wheat streak mosaic disease resistance on chromosome 8 (161.00–170.60 Mb). Using SSR markers, SNP markers, and recombination results, the *Rgsr8.1* locus was more precisely mapped to a 2.04 Mb region flanked by markers SSR-65 and SNP-25 at a physical location from 164.69 to 166.72 Mb according to the maize reference genome. Decreases in gene sequencing costs have been accompanied by increases in the use of novel genomic tools applicable for GWAS and genomic prediction (GP) for genetic analyses as well as for improving FSR resistance. For example, a GWAS-based examination of 374 maize lines for FSR resistance in three environments identified seven highly significant SNPs associated with the target trait, with five SNPs localized to chromosome 6 (168 Mb) [19]. GWAS data revealed 34 SNPs significantly associated with maize stalk rot resistance (*p* < 0.001); these SNPs were distributed on chromosomes 1, 3, 4, 6, 8, and 10, with loci on chromosomes 4 and 8 confirmed via mapped QTLs for stalk rot resistance. Several candidate genes were identified, including GRMZM2G082709 and GRMZM5G841142, which encode a protein with an NAC domain and thioredoxin reductase, respectively [10]. GWAS and GP analyses of 562 artificially inoculated tropical maize inbred lines from two populations in four environments identified 15 SNPs significantly associated with FSR resistance in both populations [28].

Although several QTLs/resistance genes have been identified, the molecular mechanisms underlying FSR resistance remain largely unexplored [29,30]. Researchers have attempted to elucidate the complex molecular mechanisms mediating maize–*Fusarium* interactions by identifying key genes on the basis of transcriptomic and metabolomic investigations, as well as functional studies [31]. Moreover, comparative metabolomics data were used to identify two metabolites (smilaside A and smiglaside C) in maize seedling roots infected with *F. graminearum*. The relative abundance of these metabolites was regulated by ethylene signaling, thereby protecting plants from this pathogen. Additionally, Sun [32] conducted a comparative transcriptomic and metabolomic analysis of *F. graminearum*-infected inbred lines with contrasting GSR resistance (i.e., resistant K09 and susceptible A08), which revealed several key hub genes potentially related to resistance to stalk rot caused by *F. graminearum*. Among the identified genes, *ZmHIR3* was one of the most important for resistance. More specifically, it was highly expressed at 48–72 h post inoculation. Notably, a mutation to this gene due to EMS resulted in increased susceptibility. Multigene analyses indicate that the JA and SA pathways are crucial for stalk rot resistance [33,34]. Several transcription factors, including *MYB*, *bHLH*, *NAC*, and *WRKY*, play key roles in influencing maize disease resistance [35]. In maize, an infection by *Fusarium* species leads to significant changes in metabolites, including anthocyanins [36]. Earlier research on disease resistance mechanisms associated with *ZmCCT* [37] and *ZmAuxRP1* [38] indicated that these two proteins have complex functions that simultaneously affect multiple processes. The coordinated effects of disease resistance-related genes and genes modulating other processes will need to be more comprehensively explored. In the current study, KASP molecular markers designed for the candidate region related to maize stalk rot resistance precisely localized *Zm00001eb260120* and *Zm00001eb260260*, which were identified as the most likely candidate genes, to chromosome 6. *Zm00001eb260120* (RING-type E3 ligase) encodes a protein with a RING domain. Interestingly, 5 transcripts, 68 orthologs, and 7 paralogs have been identified for this gene. Recent studies have shown that E3 ubiquitin ligases help regulate various disease resistance-related signaling pathways [39,40]. For example, *XA21*, which encodes a receptor-like kinase in rice, interacts with the RING-type E3 ubiquitin ligase *XB3* to regulate resistance to *Xanthomonas oryzae pv. Oryzae(Xoo*). The accumulation of *XA21* requires *XB3*, which is phosphorylated by *XA21*. *XB3* overexpression in tobacco epidermal cells induces necrosis, which is due to the E3 enzymatic activity of XB3. These findings indicate that the ubiquitin–proteasome degradation pathway is involved in *XA21*-mediated signaling associated with disease resistance [41]. *Zm00001eb260260* encodes an S-adenosyl-L-methionine-dependent methyltransferase superfamily protein. Gene-editing will need to be conducted to verify the relationship between these genes and stalk rot resistance.

Considering the problems associated with abnormal global climate change (e.g., increasingly common disastrous weather events and frequent pest and disease outbreaks), breeding excellent inbred lines is crucial for meeting the needs of highly resilient mechanized agricultural production. Disease-resistant inbred line H1710 has been used by numerous breeding institutions and companies in China. As the male parent, it has been crossed with multiple new and superior varieties currently being assessed in national or provincial variety approval trials. These crosses may contribute to the ongoing breeding of disease-resistant maize varieties.

## 4. Materials and Methods

### 4.1. Strains and Plant Materials

*F. graminearum*, which causes maize stalk rot, was provided by the Institute of Crop Sciences, Chinese Academy of Agricultural Sciences, ADFG-4 (TEF1a gene GenBank ID: MK896869. To construct the F_2_ segregating population analyzed in this study, from 2020 to 2021, Huangzaosi (a stalk rot-susceptible self-crossed line) was selected as the maternal parent for a cross with H1710 (a stalk rot-resistant self-crossed line from the Shijiazhuang Academy of Agriculture and Forestry Sciences), which served as the paternal parent. Plant materials were grown at a plant pathology nursery in Shijiazhuang, Hebei Province, China (38°05′ N, 114°52′ E), and the Zhao County Experimental Base (37°83′ N, 114°82′ E), respectively. The plants were artificially inoculated and evaluated for stalk rot resistance as previously described [42].

### 4.2. Field Resistance Evaluation

In June 2022, an F_2_ population (300 materials) was grown in an experimental field at Shijiazhuang Academy of Agriculture and Forestry Sciences (Shijiazhuang, China). Each material was individually numbered and labeled. At the seedling stage, tender leaves were collected and stored at −80 °C for the subsequent DNA extraction. Following pollen release and a 3- to 5-day interval, maize plants were inoculated via a downward-angled (45°) puncture in the stem at the second or third internode above soil level. A 1.0 mL solution of *F. graminearum* (1 × 10^6^ spores/mL) was injected directly into the stalk pith, after which puncture sites were sealed with Vaseline. The experimental field was irrigated once, which was followed by standard fertilization and irrigation management practices. During the disease development period, 1-2 irrigation cycles were completed during consecutive sunny periods to simulate rainstorm conditions followed by clear weather. *F. graminearum* was used for the artificial inoculation and subsequent evaluation of stalk rot resistance. A resistance certification report was issued for H1710. The experimental plot was 5 m long and 0.6 m wide, with 20 plants per row. Known resistant and susceptible control materials were positioned at 100-row intervals. At approximately 10 weeks post inoculation, stalks were sectioned to examine the discolored lesions in the stalk pith. The lesion area and severity were recorded for each plant, with average severity scores calculated and used to evaluate stalk rot resistance. More specifically, at 40 days post inoculation (adult plants), the inoculated internodes of individual maize plants were split and stalk rot symptom severity was assessed using scores of 0, 1, 3, 5, 7, and 9 according to an established classification standard [43].

### 4.3. BSA-Seq Analysis

Following the phenotypic analysis, 20 highly susceptible and 20 highly resistant materials were selected from the F_2_ segregating population. These materials were used for sequencing along with the parents. DNA samples were processed by Beijing Biomarker Technologies Co., Ltd, Beijing, China. For details on the reference genome used for sequencing, refer to Supplementary S1, Table S11. All DNA samples that satisfied sequencing requirements were retained for the subsequent analysis. The two DNA pools (i.e., from highly resistant and susceptible materials) and parental DNA were sequenced using the NovaSeq 6000 system (Illumina) Biomarker Technologies, Beijing, China. The generated sequencing data were used to screen for SNP and InDel loci for BSA.

### 4.4. Candidate Region Gene Annotation

The software packages BLAST (https://www.ncbi.nlm.nih.gov/) and MaizeGDB (https://maizegdb.org) were used to thoroughly annotate genes in the identified candidate region according to GO and KEGG databases.

### 4.5. KASP Marker Development

We converted SNPs polymorphically between the two parents within the candidate region into KASP markers using PolyMarker. A KASP assay was performed using a high-throughput genotyping platform (LGC, Middlesex, UK) [44].

## 5. Conclusions

This study used a segregated population obtained from a cross between the Huangzaosi susceptible inbred line and the H1710 resistant inbred line. On the basis of field disease resistance evaluations, BSA-seq analysis, and bioinformatics methods, QTLs for maize stalk rot resistance were identified. A new resistance locus (3.96 Mb) was detected on chromosome 6. KASP molecular markers were developed to more precisely localize the stalk rot resistance-related region. Candidate genes for resistance to stalk rot caused by *F. graminearum* were identified, with potential implications for further elucidating the molecular mechanisms underlying maize resistance to stalk rot following an infection by *F. graminearum*. Furthermore, the research results described herein may be relevant to exploiting available disease resistance-related genes and effective genetic resources for breeding maize varieties resistant to stalk rot. By applying both rapid breeding and conventional breeding techniques, we will accelerate the effective use of resistance QTLs or genes, create high-quality disease-resistant varieties, and lay the foundation for a molecular breeding-based system for maize resistance to stalk rot caused by *F. graminearum*.

## Figures and Tables

**Table 1:** Details regarding KASP primers.

Number	Primers	Primers Sequence	VIC	FAM
1	sn6640505	F1: GAAGGTCGGAGTCAACGGATTCAAAGGCGTCGACTCTGATAG	C	G
		F2: GAAGGTGACCAAGTTCATGCTCAAAGGCGTCGACTCTGATAC		
		R: GGAGAACATTCTGCCCCG		
2	sn6640608	F1: GAAGGTCGGAGTCAACGGATTGCACGCAGAGGCATCAGT	T	G
		F2: GAAGGTGACCAAGTTCATGCTCACGCAGAGGCATCAGG		
		R: GTGTCGAACACGTCGTTGATA		
3	sn6639872	F1: GAAGGTCGGAGTCAACGGATTCCCGTCTTATCTGGTGTCTCG	C	G
		F2: GAAGGTGACCAAGTTCATGCTCCCGTCTTATCTGGTGTCTCC		
		R: CTGCTTGTTATGTCTGCTGTCCTA		
4	sn6028745	F1: GAAGGTCGGAGTCAACGGATTCCGGCTGATTGGCCTGA	T	C
		F2: GAAGGTGACCAAGTTCATGCTCGGCTGATTGGCCTGG		
		R: CTTCCGGTCTACAGTGGCAG		
5	sn5910953	F1: GAAGGTCGGAGTCAACGGATTAAATATATAATGATTAAAGGCAAGCAC	G	A
		F2: GAAGGTGACCAAGTTCATGCTAAATATATAATGATTAAAGGCAAGCAT		
		R: GCTCAGGAAGAAGCGACCG		
6	sn6828750	F1: GAAGGTCGGAGTCAACGGATTCGCTGCAAGAAGTGGAATG	G	T
		F2: GAAGGTGACCAAGTTCATGCTCGCTGCAAGAAGTGGAATT		
		R: AACCTGTCATCCGTCGTCTT		
7	sn5630289	F1: GAAGGTCGGAGTCAACGGATTCACATTTTAATTGACAGTAAATTGATT	A	C
		F2: GAAGGTGACCAAGTTCATGCTCACATTTTAATTGACAGTAAATTGATG		
		R: AAGTGTCATCTCCAACCATTTCT		
8	sn6528233	F1: GAAGGTCGGAGTCAACGGATTATACACCTCGATCGCCCC	G	A
		F2: GAAGGTGACCAAGTTCATGCTCTATACACCTCGATCGCCCT		
		R: TGATTTCAGGGTACGAGCAGA		
9	sn6843643	F1: GAAGGTCGGAGTCAACGGATTGAGGTCTGGATCGGATGGAA	A	G
		F2: GAAGGTGACCAAGTTCATGCTGAGGTCTGGATCGGATGGAG		
		R: GCCTTTCTGCCACAATCCTT		
10	sn7185874	F1: GAAGGTCGGAGTCAACGGATTGTCGCGTACAGGGGCAC	G	A
		F2: GAAGGTGACCAAGTTCATGCTGTCGCGTACAGGGGCAT		
		R: GTCGGCGGCCATGAAC		
11	sn5955973	F1: GAAGGTCGGAGTCAACGGATTCGAGCATCCTGTCGCCA	A	G
		F2: GAAGGTGACCAAGTTCATGCTCGAGCATCCTGTCGCCG		
		R: GAATCTCGGCGGGAGTGA		

## Data Availability

The original contributions presented in this study are included in the article/Supplementary Material. Further inquiries can be directed to the corresponding author(s).

## Supplementary Materials

The following supporting information can be downloaded at: https://www.mdpi.com/article/10.3390/ijms262411866/s1.
