# Supplementary material for: Gene Mapping and Genetic Analysis of Maize Resistance to Stalk Rot
Source: Int J Mol Sci. 2025 Dec 9;26(24):11866. doi: 10.3390/ijms262411866 (PMC12732999; doi:10.3390/ijms262411866)
Supplement: Supplementary file 1 [file ijms-26-11866-s001.zip › ijms-3986721-S1.pdf]

Table S1. Descriptive Statistics for the Assessment of Sample Sequencing Data.

ID	Clean_Reads	Clean_Base	Q20(%)	Q30(%)	GC(%)
H1710	81,321,048	24,312,626,604	99.20	95.09	45.74
Huangzaosi	77,102,188	23,042,874,932	99.13	94.81	45.92
HR1	159,748,336	47,696,813,076	99.13	94.91	44.93
HS2	195,346,563	58,315,448,836	99.16	95.06	44.85

Note: **Clean_Reads**: The number of read pairs after filtering, where read1 and read2 are counted as one pair. **Clean_Base**: The total number of bases after filtering, calculated as the number of Clean_Reads multiplied by the read length. **Q20 (%)**: The percentage of bases with a Phred quality score ≥ 20. **Q30 (%)**: The percentage of bases with a Phred quality score ≥ 30. **GC (%)**: The GC content of the sample, representing the percentage of G and C bases among the total bases.

Table S2. Descriptive Statistics for the Assessment of Sample Sequencing Data.

ID	Total_reads	Mapped(%)	Properly_mapped(%)
H1710	162,642,096	99.53	88.54
Huangzaosi	154,204,376	99.26	88.46
HR1	319,496,672	97.43	79.74
HS2	390,693,126	99.23	82.68

Note: **Total_Reads:** The total number of clean reads for the sample. Paired-end reads are counted separately, meaning read1 and read2 are counted as two individual reads. **Mapped (%)**: The percentage of clean reads aligned to the reference genome. **Properly_mapped (%)**: The percentage of read pairs where both ends are correctly aligned to the reference genome with an insert size conforming to the expected library fragment length distribution.

Table S3. Statistics on Sample Coverage Depth and Coverage Proportion.

ID	Ave_depth	Cov_ratio_1X(%)	Cov_ratio_5X(%)	Cov_ratio_10X(%)
H1710	10	88.14	72.88	49.92
Huangzaosi	10	87.52	70.5	45.09
HR1	20	94.64	85.31	73.74
HS2	26	95.19	87.39	78.7

Note: Ave_depth: Average sequencing depth of the sample. **Cov_ratio_?X (%)**: The percentage of bases in the reference genome covered at or above the specified depth threshold (?X).

Table S4. Distribution of SNP Types Categorized by Sample.

ID	SNPnumber	Transition	Transversion	Ti/Tv	Heterozygosity	Homozygosity	Het-ratio
H1710	6,268,510	4,484,432	1,784,078	2.51	1,638,824	4,629,686	26.14%
Huangzaosi	6,829,194	4,882,191	1,947,003	2.5	1,739,365	5,089,829	25.46%
HR1	9,458,666	6,763,093	2,695,573	2.5	6,158,829	3,299,837	65.11%
HS2	9,793,836	7,010,592	2,783,244	2.51	6,492,799	3,301,037	66.29%
Total	11,271,056	8,089,704	3,181,352	2.54	-	-	

Table S5. Statistical Table of Genes with Non-synonymous and Frameshift Mutations in Candidate Regions.

NO	gene	Gene expression	Pfam_annotation	Swissprot_annotation	TrEMBL_annotation	mutation type
1	Zm00001eb260200	up	PHD-finger	PHD finger protein EHD3 OS=Oryza sativa subsp.	RING/FYVE/PHD-type zinc finger family protein	Non synonymous mutation
2	Zm00001eb260120	up	Ring finger domain	Receptor homology region, transmembrane domain- and RING domain-containing protein 1	Receptor homology region transmembrane domain-and RING domain-containing protein 2	Non synonymous mutation

NO	gene	Gene expression	Pfam_annotation	Swissprot_annotation	TrEMBL_annotation	mutation type
3	Zm00001eb260190	up	Triose-phosphate Transporter family	UDP-galactose transporter 1	GDP-mannose transporter GONST5	Non synonymous mutation
4	Zm00001eb260280	up	Plant invertase/pectin methylesterase inhibitor	Pectinesterase inhibitor 28	Cell wall/vacuolar inhibitor of fructosidase 2	Non synonymous mutation
5	Zm00001eb260260	up	Putative S-adenosyl-L-methionine-dependent methyltransferase	--	S-adenosyl-L-methionine-dependent methyltransferase superfamily protein	Non synonymous mutation
6	Zm00001eb259990	up	F-box domain	--	Retrovirus-related Pol polyprotein from transposon TNT 1-94	Non synonymous mutation
7	Zm00001eb260100	up	YT521-B-like domain	YTH domain-containing protein ECT2	Evolutionarily conserved C-terminal region 7	Non synonymous mutation
8	Zm00001eb259960	up	Heavy-metal-associated domain	Heavy metal-associated isoprenylated plant protein 20	Farnesylated protein 2	Non synonymous mutation
9	Zm00001eb260210	up	YL1 nuclear protein	SWR1 complex subunit 2	Vacuolar protein sorting protein 72	Non synonymous mutation
10	Zm00001eb260170	up	Phosphatidylinositol-4-phosphate 5-Kinase	Putative 1-phosphatidylinositol-3-phosphate 5-kinase FAB1D	1-phosphatidylinositol-3-phosphate 5-kinase	Non synonymous mutation
11	Zm00001eb260140	up	2Fe-2S iron-sulfur cluster binding domain	Ferredoxin-1, chloroplastic	Ferredoxin	Non synonymous mutation
12	Zm00001eb259890	up	XPG I-region	Single-strand DNA endonuclease 1	Flap endonuclease GEN-like 2	Non synonymous mutation
13	Zm00001eb259970	up	VQ motif	Protein MKS1	Protein MKS1	Non synonymous mutation
14	Zm00001eb260060	up	--	--	Uncharacterized protein	Non synonymous mutation
15	Zm00001eb260090	up	Protein of unknown function (DUF679)	Protein DMP3	DUF679 domain membrane protein 7	Non synonymous mutation
16	Zm00001eb260230	up	Shwachman-Bodian-Diamond syndrome (SBDS) protein	--	Shwachman-Bodian-Diamond syndrome protein	Non synonymous mutation
17	Zm00001eb260240	up	Triose-phosphate Transporter family	GDP-mannose transporter GONST1	GDP-mannose transporter GONST1	Non synonymous mutation
18	Zm00001eb260040	up	PPR repeat	Pentatricopeptide repeat-containing protein At2g33760	Pentatricopeptide repeat-containing protein	Non synonymous mutation
19	Zm00001eb260000	up	No apical meristem (NAM) protein	NAC domain-containing protein 30	Putative NAC domain transcription factor superfamily protein	Non synonymous mutation
20	Zm00001eb260130	up	--	SEED MATURATION PROTEIN 1	Seed maturation protein	Non synonymous mutation
21	Zm00001eb260100	up	YT521-B-like domain	YTH domain-containing pro-	Evolutionarily conserved C-terminal re-	frameshift mutation

NO	gene	Gene expression	Pfam_annotation	Swissprot_annotation	TrEMBL_annotation	mutation type
				tein ECT2	gion 7	
22	Zm00001e 029130	up	Phosphatidylinositol-4-phosphate 5-Kinase	Putative 1-phosphatidylinositol-3-phosphate 5-kinase FAB1D	1-phosphatidylinositol-3-phosphate 5-kinase	frameshift mutation
23	Zm00001e 029136	up	Triose-phosphate Transporter family	GDP-mannose transporter GONST1	GDP-mannose transporter GONST1	frameshift mutation

Table S6. Statistical Summary of SNP Annotation Results within the Candidate Region.

Type	Huangzaosi vs. H1710	HS2 vs. HR1
UTR_5_PRIME	34	26
UTR_3_PRIME	41	32
UPSTREAM	396	293
SYNONYMOUS_CODING	99	54
STOP_GANED	1	0
START_LOST	1	1
START_GAINED	2	0
SPLICE_SITE_REGION	5	3
NON_SYNONYMOUS_CODING	87	48
INTRON	207	116
INTERGENIC	7,594	4,968
DOWNSTREAM	513	325
Other	0	0

Table S7. Statistical Summary of InDel Annotation Results within the Candidate Region.

Type	Huangzaosi vs. H1710	HS2 vs. HR1
UTR_5_PRIME	14	7
UTR_3_PRIME	34	26
UPSTREAM	83	74
STOP_GANED	0	1
SPLICE_SITE_REGION	1	1
INTRON	60	40
INTRAGENIC	1	1
INTERGENIC	651	487
FRAME_SHIFT	5	4
DOWNSTREAM	116	9
CODON_INSERTION	4	2
CODON_DELETION	3	2
CODON_CHANGE_PLUS_CODON_INSERTION	2	2
CODON_CHANGE_PLUS_CODON_DELETION	1	2
Other	0	0

Table S8. Summary of Functional Annotation Results for Genes Harboring SNPs and InDels in the Candidate Region.

Annotated _databases	Gene Num	Non_Syn Gene Num	FRAME_SHIFT Gene Num
NR	37	21	3
NT	38	21	3
trEMBL	38	21	3
SwissProt	28	16	3
GO	33	20	3

KEGG	24	15	3
COG	11	5	1
Total	38	21	3

Table S9. Graphical Representation of topGO Enrichment Analysis for Genes Corresponding to SNPs in the Candidate Region.

GO.ID	Term	Annotated	Significant	Expected	KS
GO:0005886	plasma membrane	1,657	1	2.17	0.016
GO:0005634	nucleus	4,527	7	5.93	0.033
GO:0005783	endoplasmic reticulum	623	1	0.820	0.044
GO:0016020	membrane	7,831	9	10.25	0.064
GO:0031967	organelle envelope	541	2	0.710	0.070
GO:0031975	envelope	541	2	0.710	0.070
GO:0044432	endoplasmic reticulum part	358	1	0.470	0.070
GO:0005789	endoplasmic reticulum membrane	319	1	0.420	0.071
GO:0098589	membrane region	336	1	0.440	0.089

Note: GOID: Identifier of the GO term. **Term**: Name of the GO term. **Annotated**: Number of genes annotated to the specific GO term across the background set. **Significant**: Number of genes within the associated region annotated to the specific GO term. **Expected**: Expected number of genes annotated to the GO term. **KS**: Kolmogorov-Smirnov statistic indicating the enrichment significance; a smaller KS value denotes a more significant enrichment.

Table S10. Statistical Table of Partial KEGG Enrichment Results for Genes Corresponding to SNPs in the Candidate Region.

Pathway	KO	Enrichment_Factor	Q-value
Photosynthesis	ko00195	16.25	0.003
Glycine,serine and threonine metabolism	ko00260	12.16	0.045
Glyoxylate and dicarboxylate metabolism	ko00630	9.77	0.069
Carbon metabolism	ko01200	3.56	0.424

Note: **Pathway:** KEGG pathway name; **KO**: KEGG orthology identifier;.**Q_value**: Statistical significance of enrichment, where a smaller value indicates a higher degree of enrichment.

Table S11. Descriptive Statistics for the Reference Genome.

Reference Genome	Genome Size(Mb)	GC(%)	Assembly
Zea_mays:Zm_B73_REFERENCE_NAM_5.0	2183	46.63	Chromosome
