# Supplementary material for: Gene Mapping and Genetic Analysis of Maize Resistance to Stalk Rot
Source: Int J Mol Sci. 2025 Dec 9;26(24):11866. doi: 10.3390/ijms262411866 (PMC12732999; doi:10.3390/ijms262411866)
Supplement: Supplementary file 1 [file ijms-26-11866-s001.zip › ijms-3986721-S2.pdf]

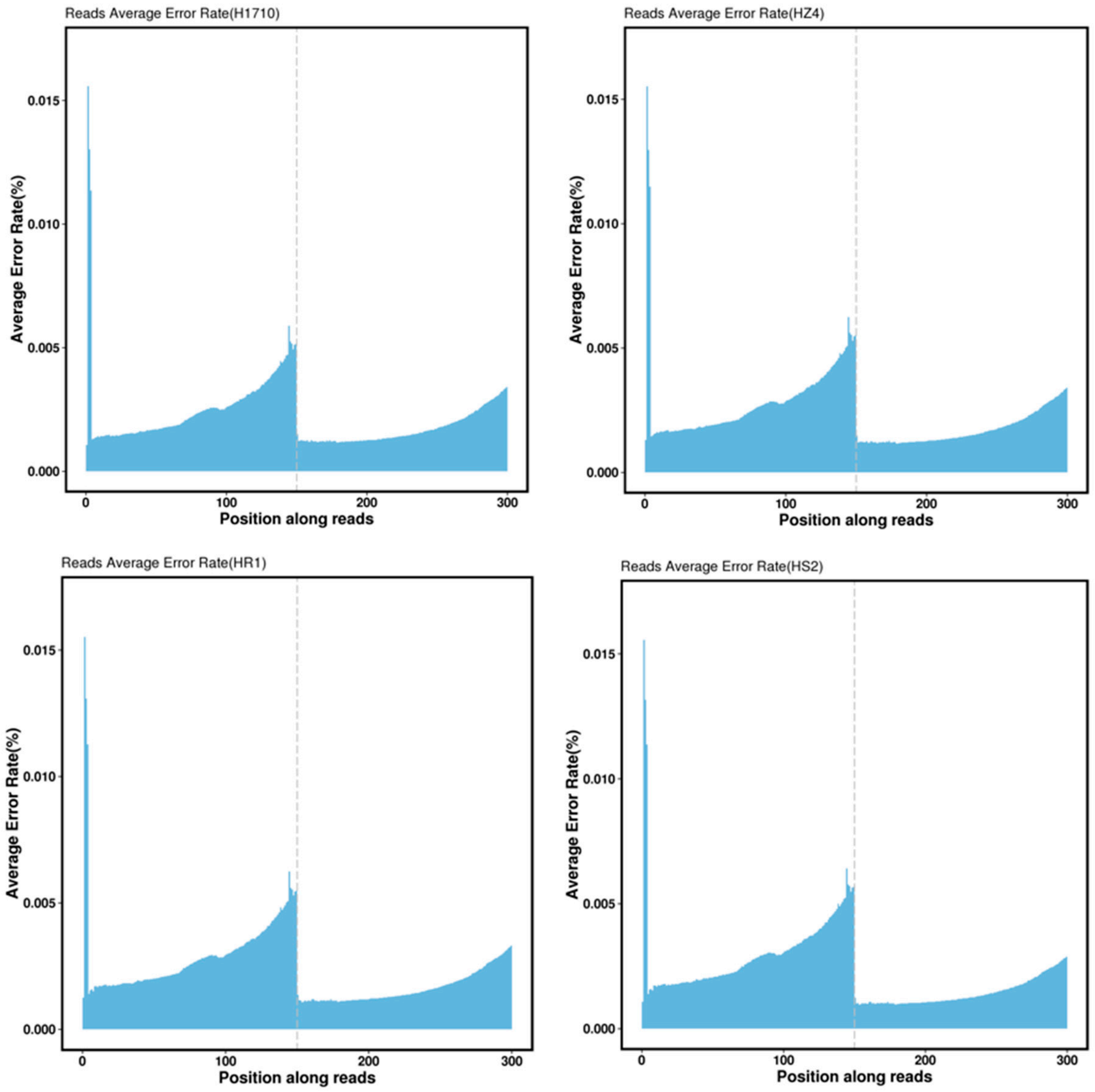

Figure S1. Based on the relationship between quality values and error rates, we converted the quality values into error rates and generated the error rate distribution plot as follows:.

Note: HZ4 is Huangzaosi; HR1 and HS2 are designated as the highly disease-resistant and highly disease-susceptible groups, respectively.

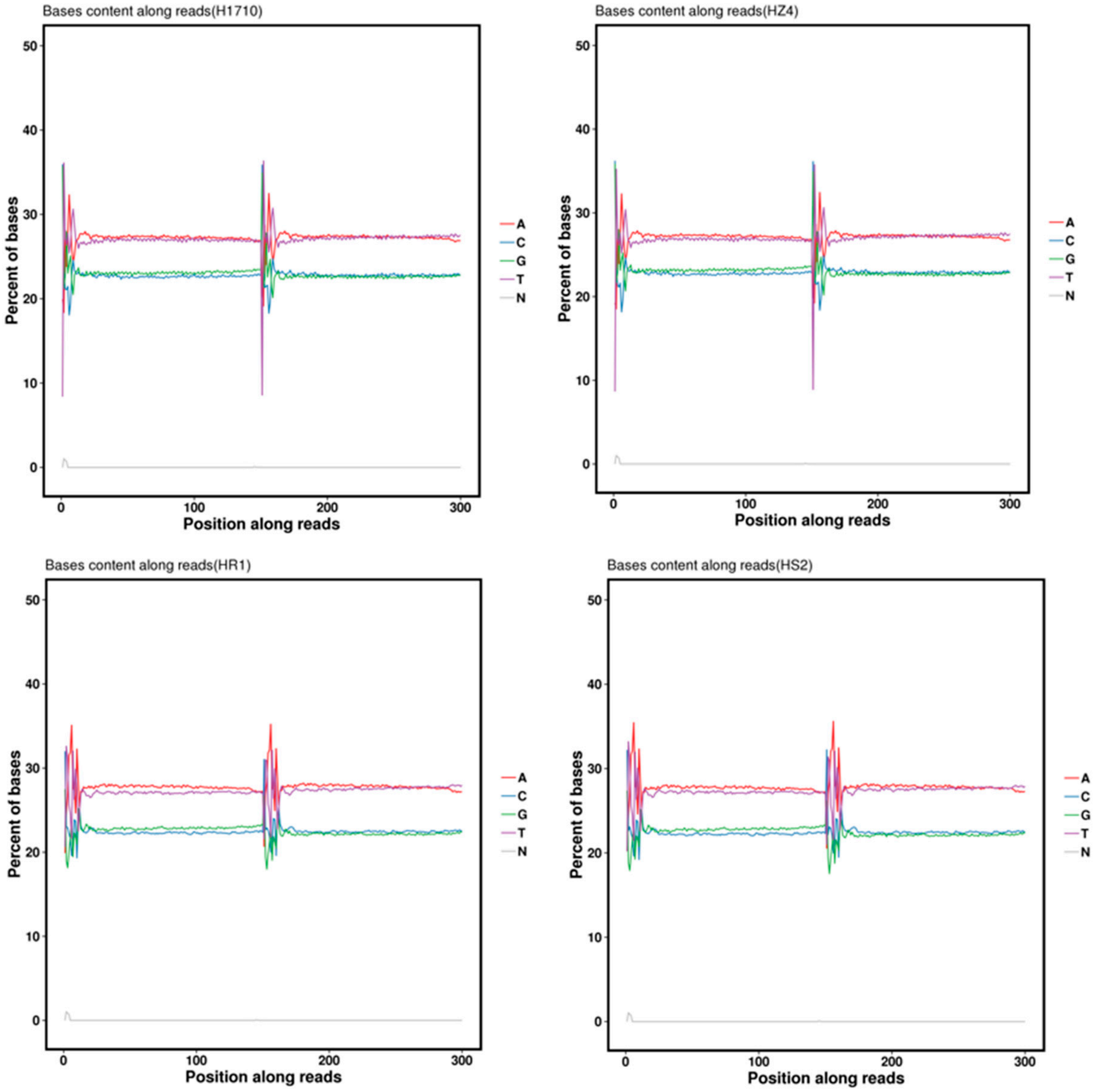

Figure S2. The proportional distribution of each base in the sample is illustrated.

Note: The *x*-axis represents the base position in the reads, and the *y*-axis represents the proportion of each base. Different colors represent different base types: green for base G, blue for base C, red for base A, purple for base T, and gray for base N, which is an unrecognized base during sequencing. The first 150 bp show the base distribution of the first read in paired-end sequencing, while the last 150 bp represent the base distribution of the other read. Each cycle corresponds to a sequenced base position. For example, the first cycle indicates the distribution of A, T, G, C, and N at the first base position across all sequencing reads in the project. HZ4 is Huangzaosi; HR1 and HS2 are designated as the highly disease-resistant and highly disease-susceptible groups, respectively.

The results from the graph indicate that the AT and GC bases show essentially no separation, and the curves are relatively flat, suggesting normal sequencing results.

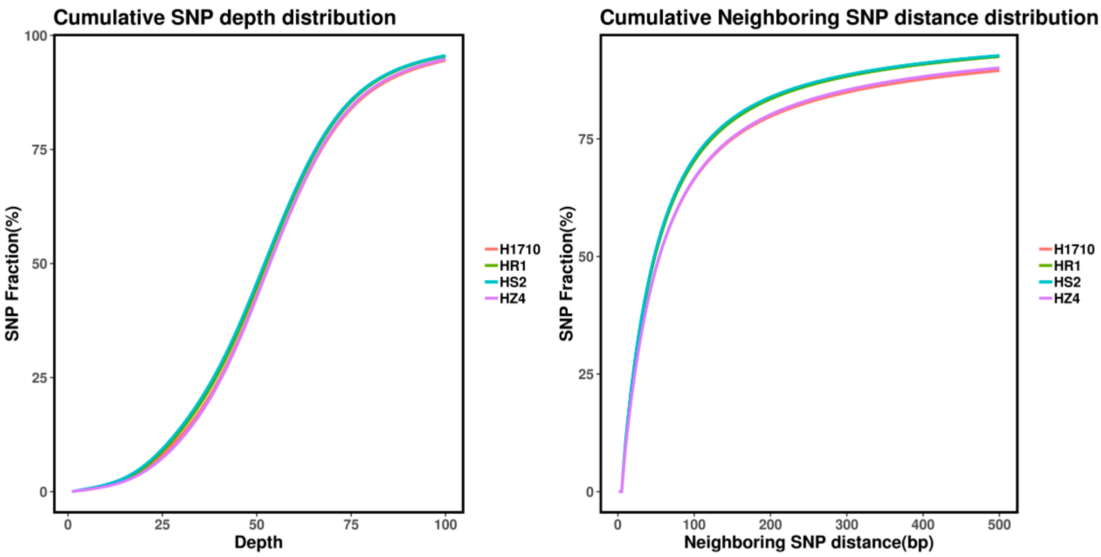

Figure S3. To evaluate the confidence of the called SNPs, we generated cumulative distribution plots for the read depth supporting each SNP and the physical distances between neighboring SNPs.

Note: HZ4 is Huangzaosi; HR1 and HS2 are designated as the highly disease-resistant and highly disease-susceptible groups, respectively.

The left plot depicts the cumulative distribution of read support for SNPs, while the right plot depicts the cumulative distribution of distances between successive SNPs.

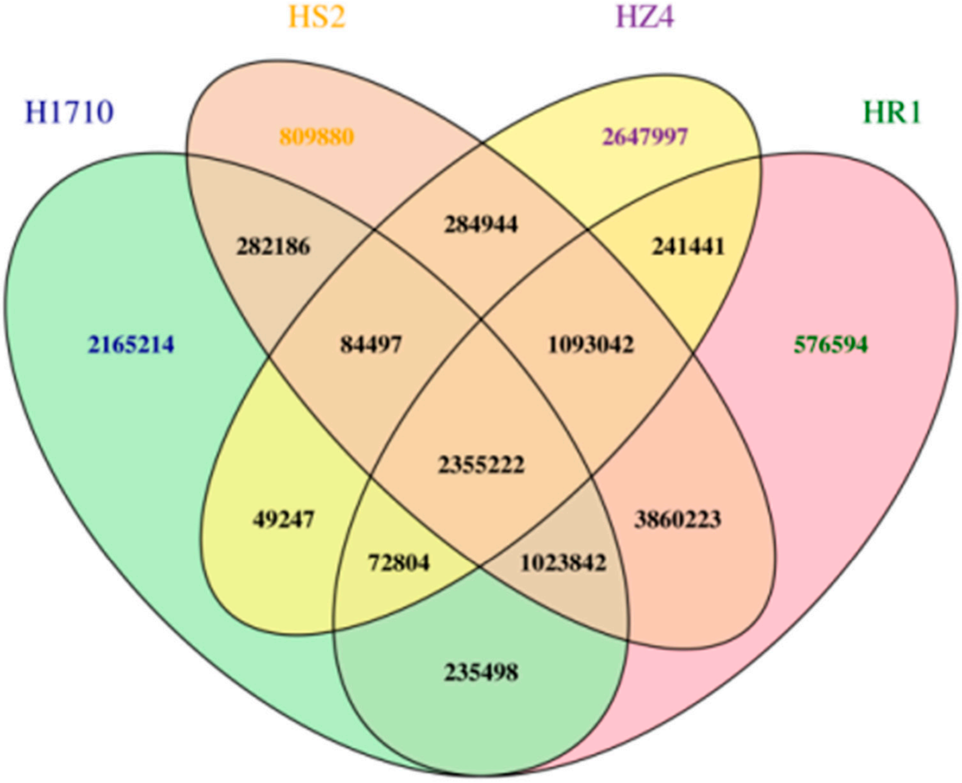

Figure S4-1. The Venn diagram depicts the common and sample-specific single nucleotide polymorphisms (SNPs) identified across the sample:.

Note: HZ4 is Huangzaosi; HR1 and HS2 are designated as the highly disease-resistant and highly disease-susceptible groups, respectively.

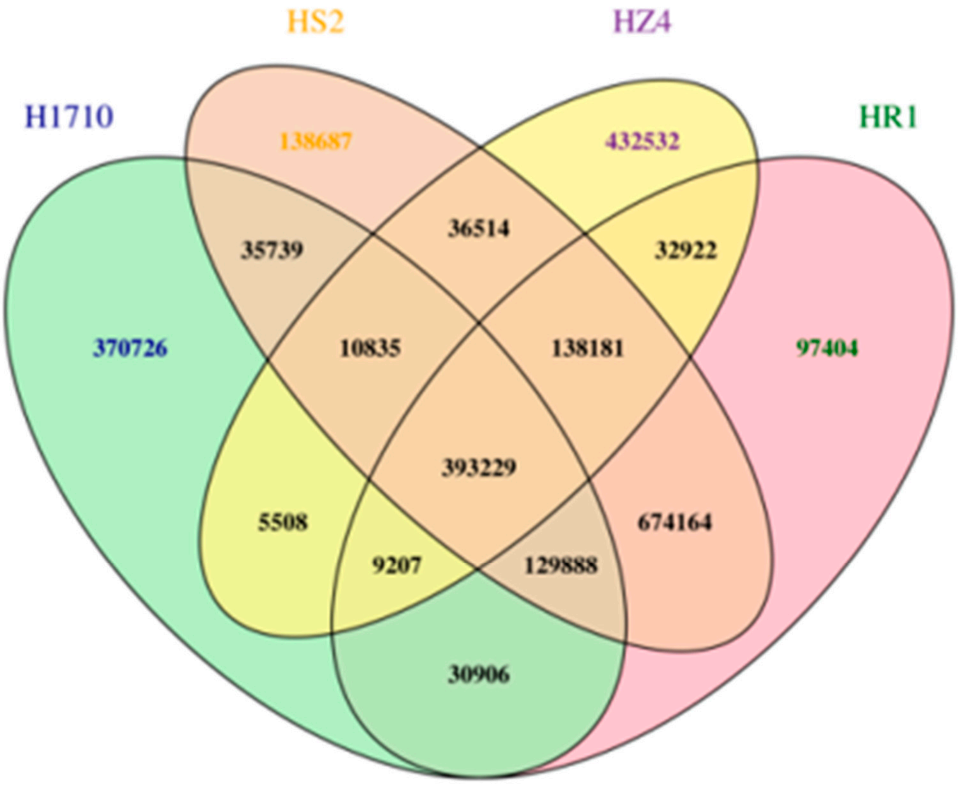

Figure S4-2. The Venn diagram depicts the common and sample-specific InDels identified across the samples:.

Note: HZ4 is Huangzaosi; HR1 and HS2 are designated as the highly disease-resistant and highly disease-susceptible groups, respectively.

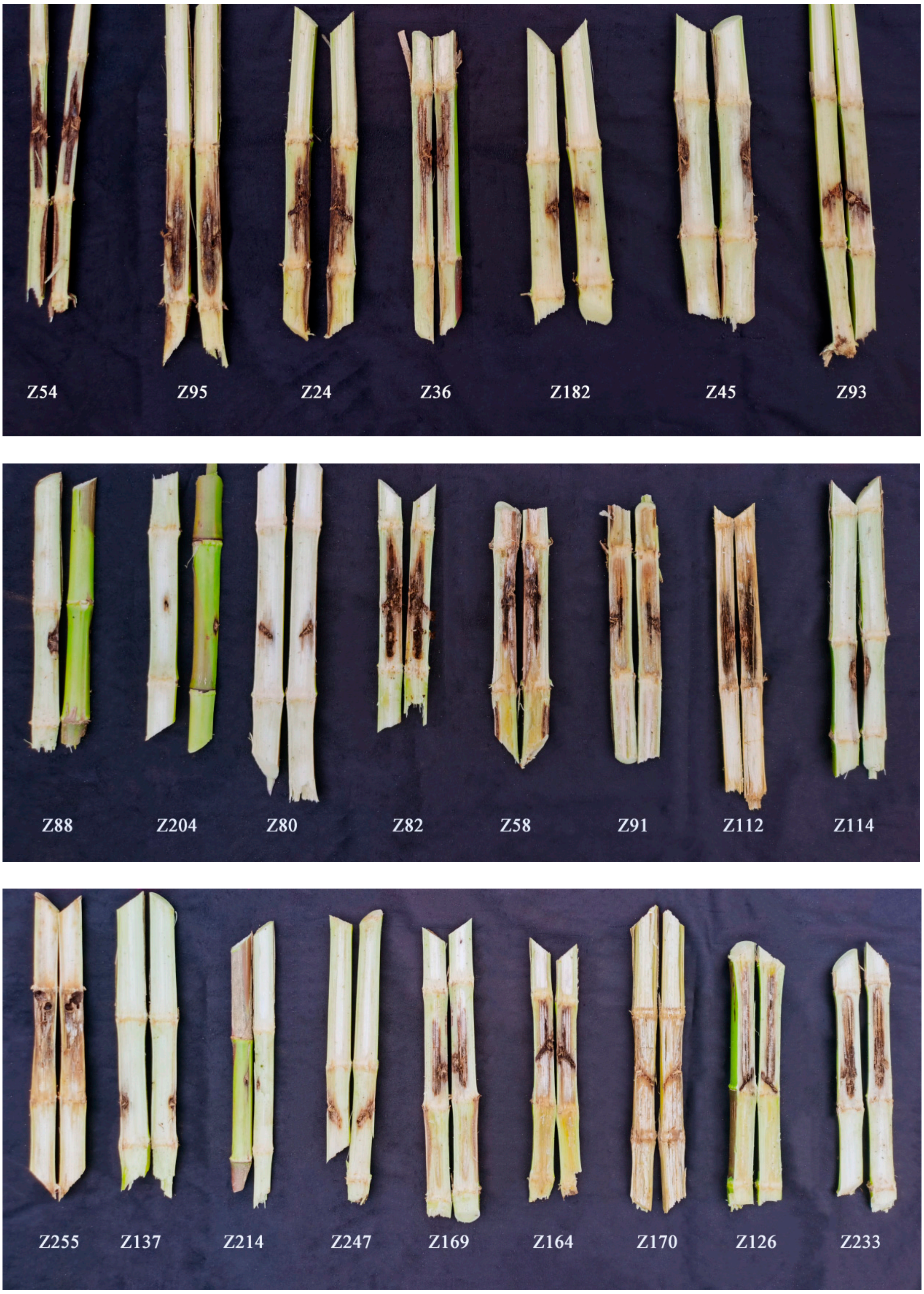

Figure S5. Field survey images of the $F_{2:3}$ population.
